# Plasma-Modified PI Substrate for Highly Reliable Laser-Sintered Copper Films Using Cu_2_O Nanoparticles

**DOI:** 10.3390/nano12183237

**Published:** 2022-09-18

**Authors:** Wei-Han Cheng, Ming-Tsang Lee, Kiyokazu Yasuda, Jenn-Ming Song

**Affiliations:** 1 Department of Materials Science and Engineering, National Chung Hsing University, Taichung 402, Taiwan; 2 Department of Power Mechanical Engineering, National Tsing Hua University, Hsinchu 30013, Taiwan; 3 Division of Materials and Manufacturing Science, Graduate School of Engineering, Osaka University, Osaka 565-0871, Japan; 4 Innovation and Development Center of Sustainable Agriculture, National Chung Hsing University, Taichung 402, Taiwan; 5 Smart Sustainable New Agriculture Research Center, National Chung Hsing University, Taichung 402, Taiwan

**Keywords:** plasma modification, polyimide, surface properties, laser sintering, cuprous oxide

## Abstract

Plasma modification of polyimide (PI) substrates upon which electrical circuits are fabricated by the laser sintering of cuprous oxide nanoparticle pastes was investigated systematically in this study. Surface properties of the PI substrate were investigated by carrying out atomic force microscopy (AFM) and X-ray photoelectron spectroscopy (XPS), and contact angle measurements. Experimental results show that surface characteristics of PI substrates, including surface energy, surface roughness, and surface binding significantly affected the mechanical reliability of the sintered copper structure. Among the plasma gases tested (air, O_2_, Ar-5%H_2_, and N_2_-30%H_2_), O_2_ plasma caused the roughest PI surface as well as the most C=O and C–OH surface binding resulting in an increased polar component of the surface energy. The combination of all those factors caused superior bending fatigue resistance.

## 1. Introduction

The demand for flexible circuit electronics such as those used on display panels, radio frequency identification devices, thin-film transistors, and organic solar cells is increasing [1,2]. The most commonly used flexible substrates are papers and polymers, which have stringent requirements regarding processing temperatures. To correspond with the roll-to-roll process of flexible substrate and to reduce heating time, several sintering techniques have been investigated including: laser sintering, rapid electrical sintering, and pulsed light sintering [3,4]. The use of continuous-wave or pulsed laser for the sintering of metal inks or paste has attracted much attention. Previous studies have shown that there are two mechanisms involved in the laser sintering of metal particles [5]. The first is a photothermal effect, the energy absorbed from the light beam by the metal particles causes the atoms in the crystal lattice to vibrate and the acceleration produces heat. The second mechanism is the formation of surface plasma on the metal surface results in surface plasma resonance [6] which also affect the absorption of laser beam energy by the particles. In addition, the laser sintering of particles are influenced by the multiple reflection and absorption effect in the particle films that can be interpreted by the optical penetration depth (OPD). The OPD depends on several parameters of the particle film including the surface morphology of the particles, the degree of particle smoothness, and the gaps between them, etc. Fischer et al. [7,8] carried out laser sintering of titanium metal powder with grain sizes of 8–30 μm. An OPD of 65 μm, which is much higher than the OPD of the bulk material, was achieved. This result suggests that by tuning the material properties and lase parameters, 3D manufacturing of microstructures by laser sintering is feasible.

Cost efficiency, excellent thermal compatibility, and good electrical conductance resulted in circuits made by the photo-sintering of copper nanoparticles (NPs) becoming an attractive technology and research topic to the application of nanometals in microelectronics. For instance, by using a commercial nano-copper ink (5nm copper NPs in 2-methoxy ethanol) and a 50 J/cm^2^ energy flash light for sintering, a sintered copper specimen with resistivity as low as the bulk material (5 μΩ-cm) was reached [9]. A later study reported by the same group [10] indicated that the presence of an oxide surface layer on the nano-copper particles is inevitable. This layer affects not only the conductivity of the sintered film but also the diffusion of copper during sintering. To achieve one-step photoreduction/sintering, one solution is to select solvents or additives that can be photoactivated. Ethylene glycol and polyvinylpyrrolidone (PVP) can be added to the copper inks to cause photodissociation under high-energy pulse flash. Formic acid or ethanol containing hydroxy groups can also be used as reducing agents. Laser sintering of nano-copper particles into a highly conductive thin film that has a compatible resistivity as that of bulk copper has also been reported [11,12,13]. In addition to the elimination of organic residues, the laser energy density, scanning pitch, and scanning speed have considerable influences on the oxidation and resistivity of the sintered films. Good conductivity can be achieved only when nano-copper particles can fuse completely, grains can grow, and oxidation can be prevented.

Although it is feasible to attain copper films with good conductivities from sintering nano-copper particles, synthesizing and handling nano-copper particles remain challenging since nano-copper particles actively react with oxygen. The use of oxygen-proof surfactants usually leads to decreased electrical conductivity due to incomplete removal during the sintering process. To overcome the difficulty of storing copper nanoparticles, cupric oxide (CuO) NPs which can be converted to Cu particles and fused to form a connected conductive line through photonic sintering have been well-developed [14,15,16]. In the current study, a cuprous oxide (Cu_2_O) NP paste that is suitable for laser sintering technology is be developed to form circuits on polyimide substrate (PI). The raw material used, Cu_2_O NPs, is more easily reduced than the commercial CuO NPs in an earlier study [17].

**Figure 1:**
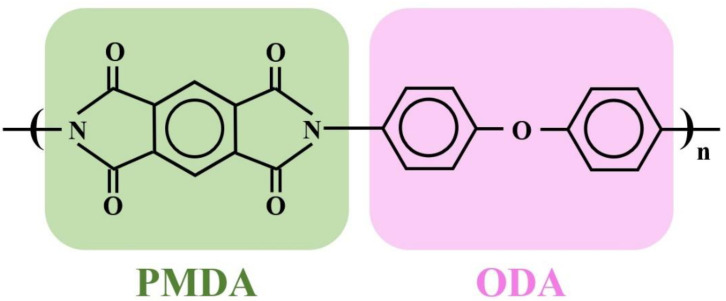
Structural formulas of PI.

In order to enhance adhesive strength between polymeric substrates and metals (e.g., sputtered Cu/polyimide (PI) [18] and Ti sheets/PI [19]), air plasma and atmospheric pressure plasma were applied to modify the substrate surface. Inspired by this, plasma modifications of the PI surface are carried out for the purpose to improve bending fatigue resistance of the laser sintered copper film. Four gases were tested for the plasma treatment: air, O_2_, Ar-5%H_2_, and N_2_-30%H_2_. Surface properties of the PI substrate, of which the structural formula has two parts, PMDA and ODA [20], see Figure 1, were measured by using atomic force microscopy (AFM) and X-ray photoelectron spectroscopy (XPS) to investigate the mechanism behind the enhancement of the mechanical properties of the copper sintered structure/polymeric interface.

## 2. Experimental Procedures

### 2.1. Plasma Modification of the Surface of a Polymeric Substrate

The polymeric substrate used in this study was a polyimide (PI) sheet with 50 μm in thickness. The film was cut into 4 × 3 cm^2^ pieces and the surface was cleaned by ethanol, acetone, and DI water, respectively. The samples were placed in the plasma chamber (Ceres Crown 1303, Ceres Energy Industry Inc., Hexaham, UK) and treated using gas sources of O_2_, Ar-5%H_2_, and N_2_-30%H_2_ individually. The gas flow rate used was 50 sccm, plasma power was fixed at 29.6 W, chamber pressure was 0.5 torr, and processing time was between 10 and 30 min. A comparison with air plasma was also conducted.

This study used contact angles to estimate the surface energy ($\gamma_{s}$) which has two components, dispersive surface energy $\gamma_{s}{}^{D}$ and polarized surface energy $\gamma_{s}{}^{p}$. Measurements were taken using deionized water ($\gamma_{l}{}^{P}$ = 46.8 mN/m and $\gamma_{l}{}^{D}$ = 26 mN/m) and CH_2_I_2_ ($\gamma_{l}{}^{P}$ = 6.7 mN/m, $\gamma_{l}{}^{D}$ = 44.1 mN/m), based on Equations (1) and (2) [21].

(1) $$\gamma_{sl}=\gamma_{s}-\gamma_{l}\cos\theta$$

(2) $$\gamma_{sl}=\gamma_{s}+\gamma_{l}-2\left[{\left(\gamma_{s}^{D}+\gamma_{l}^{D}\right)}^{\frac{1}{2}}+{\left(\gamma_{s}^{P}+\gamma_{l}^{P}\right)}^{\frac{1}{2}}\right]$$

### 2.2. Preparation and Coating with Cuprous Oxide Composite Pastes

Procedures of Cu_2_O nanoparticle synthesis were described as follows: 2.5 g CuCl_2_ was added into 300 mL DI water to form CuCl_2_ solution, 30 mL of 1M NaOH solution was then mixed with the above solution under constant magnetic stirring. PVP and N_2_H_4_ were then added in sequence to obtain Cu_2_O nanoparticles. Herein PVP was the surfactant, while N_2_H_4_ was the reductant.

**Figure 2:**
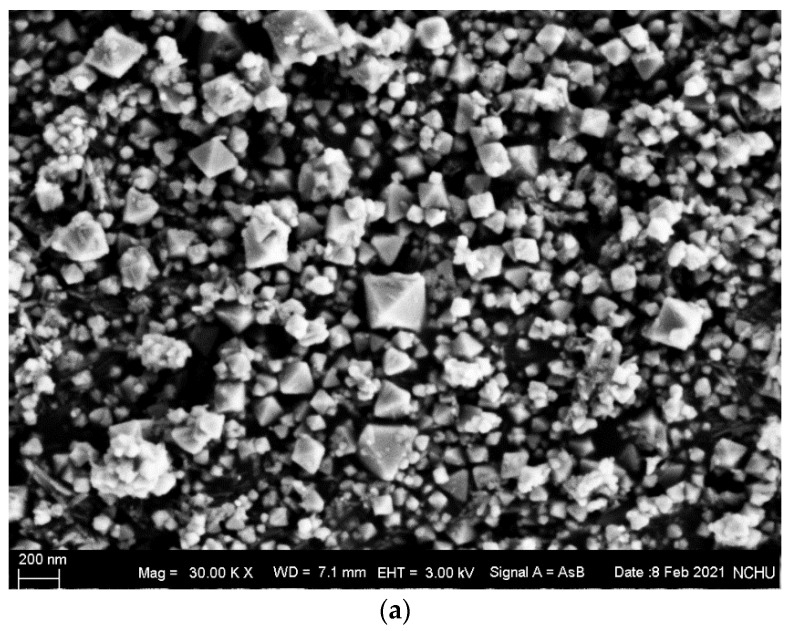

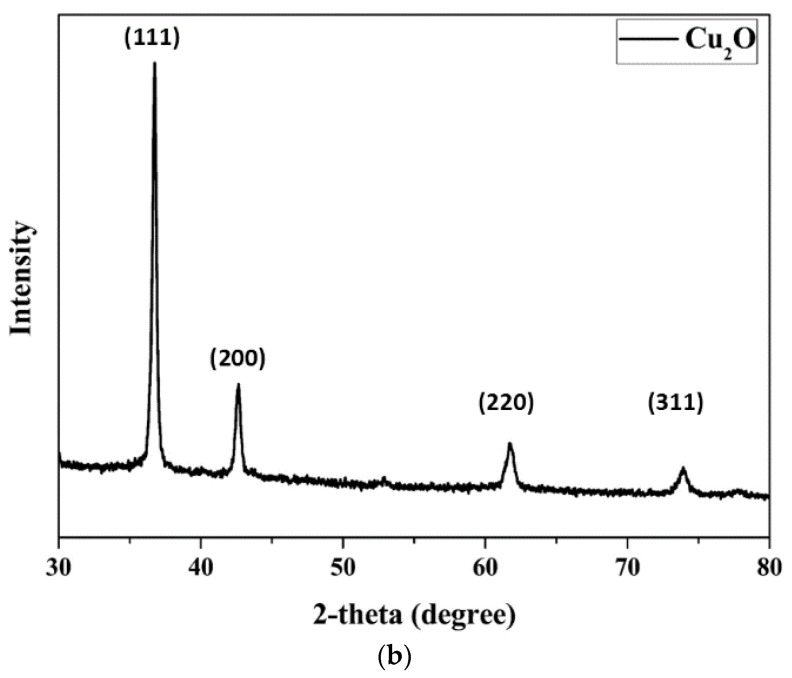
Cuprous oxide nanoparticles: (**a**) SEM image, and (**b**) XRD pattern.

The SEM image in Figure 2a shows that the mean size of the Cu_2_O grains was around 200 nm, and the maximum grain size did not exceed 400 nm. The structural phase was identified based on the XRD pattern shown in Figure 2b. The solvent used for paste preparation was α-terpineol. PVP was used for thickening, and formic acid as a reduction agent. Because previous photonic sintering studies had shown that copper formate enhanced the continuity of copper sintered structures [17], copper formate was used in this study as an additive to some of the pastes. The screen-printing method was used to coat the composite paste evenly onto the PI polymeric substrate.

### 2.3. Laser Sintering and Reliability Testing

A continuous-wave diode laser, (G5F27-1000P, MeshTel Intelite Inc., Genoa, NV, USA) with a wavelength of 638 nm and a maximum power of 1000 mW, was used. The controlled processing parameters were: laser power, scan speed, and scan distance. The laser beam was guided by a galvanometer scanning system and a predetermined diagram was followed. The final sintered product was cleaned with ethanol to remove unreacted paste.

**Figure 3:**
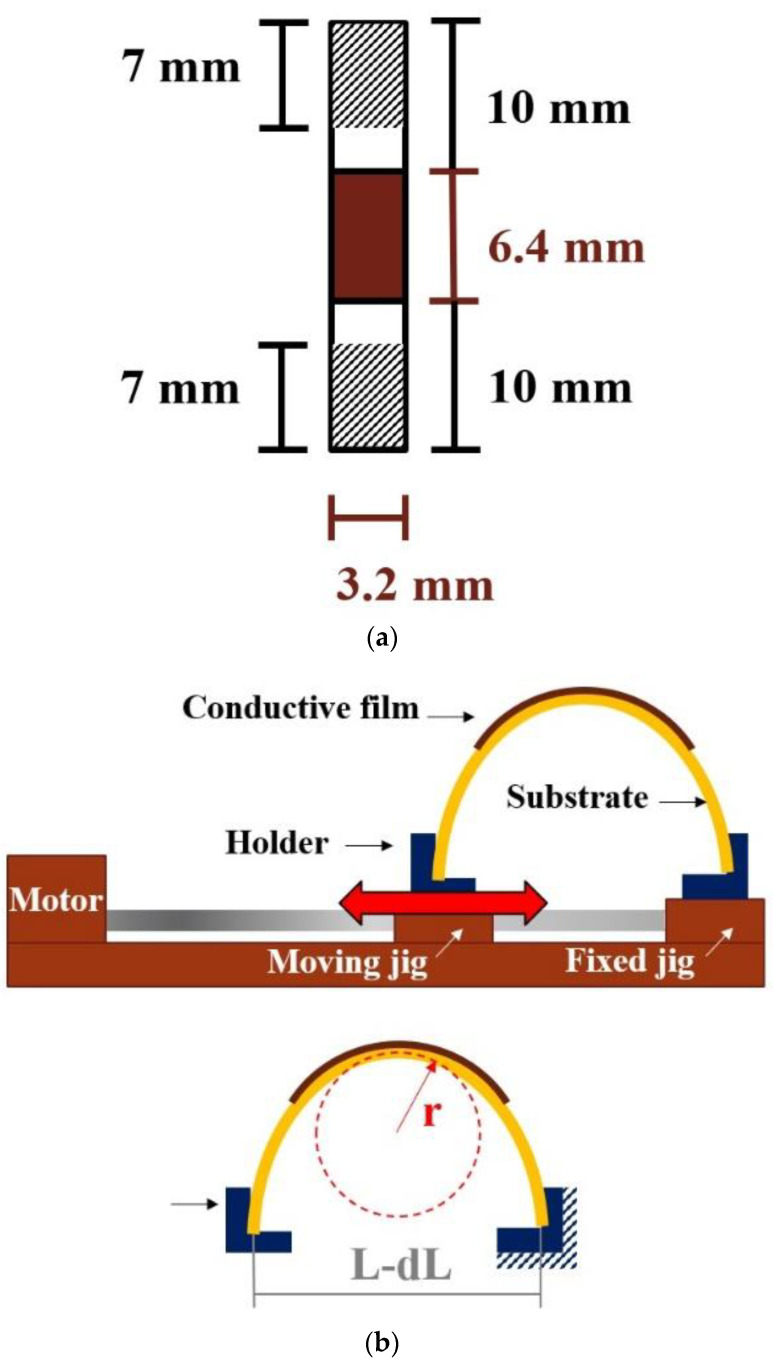
Schematic of the bending experiment: (**a**) test sample and (**b**) apparatus setup.

The mechanical reliability of the sintered films was determined by a bending fatigue test. The PI specimens were cut to size, see Figure 3a,b for testing. The testing condition was a fixed radius of curvature (*R_nom_*: 2.91 mm) with a bending frequency of 1 Hz. Changes in resistivity as a function of the bending cycle were recorded. The radius of curvature was calculated based on Equation (3) as follows [22]:

(3) $$R_{nom}=\frac{L}{2\pi \sqrt{\frac{dL}{L}-\frac{\pi^{2}h_{s}{}^{2}}{12L^{2}}}}$$

where *R_nom_* is the radius of curvature, *L* is the initial length of the substrate before bending, *dL* is the length difference before and after substrate bending, and *h_s_* is substrate thickness. Resistivity failure was defined as an increase in resistivity to double the original value.

## 3. Results and Discussion

### 3.1. Optimal Sintering Conditions

**Figure 4:**
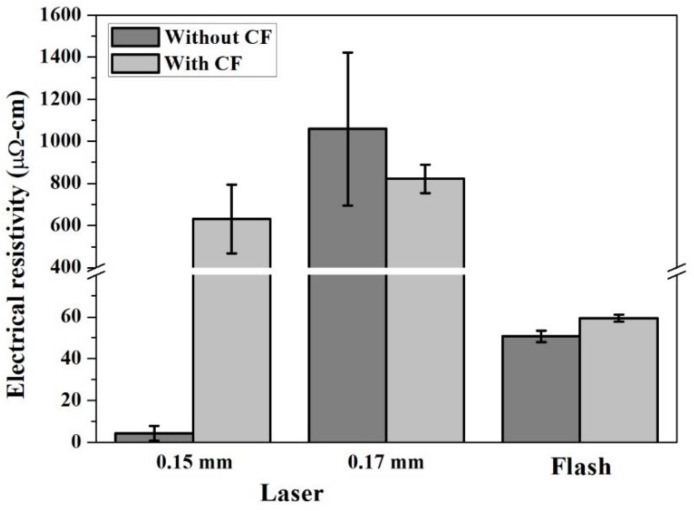
Electrical resistivity of sintered structure using laser and pulsed flash respectively (CF: copper formate).

**Figure 5:**
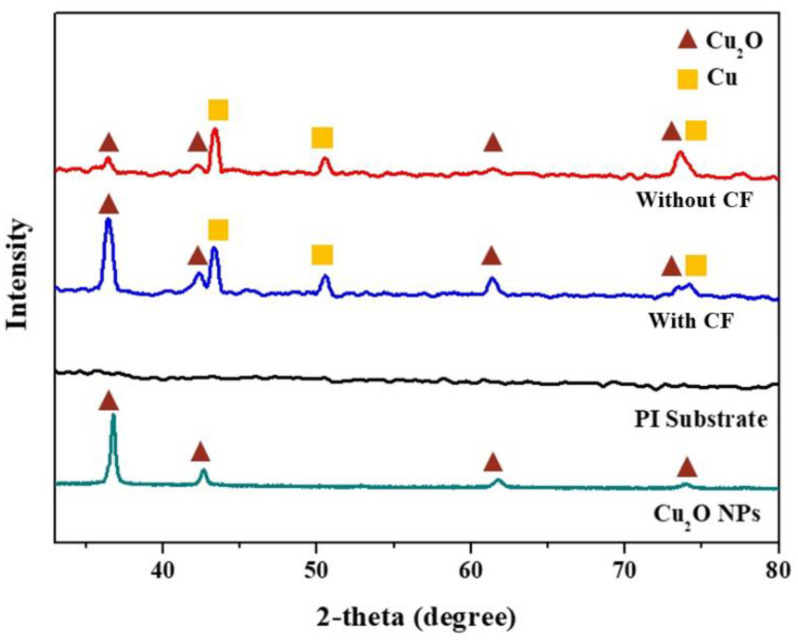
XRD patterns of laser-sintered structure, Cu_2_O nanoparticles, and PI substrate (CF: copper formate).

Sintering of the Cu_2_O paste formed a thin copper conductive layer on the PI substrate. Figure 4 shows that at 300 mW, a scanning speed of 2 mm/s and a scanning pitch of 0.15 mm, a layer with a mean resistivity approximately 4 μΩ-cm can be obtained (the theoretical resistivity of pure copper is 1.8 μΩ-cm). The results were as good as, or better than, the resistivity reported in the literature [13,23,24]. The window of the processing conditions with good results was small. Changing the scanning hatch to 0.17 mm, adding copper formate, or using a xenon flash light source all caused the resistivity to increase dramatically. Figure 5 illustrates the XRD patterns of the sintered structures subject to optimal laser irradiation, as well as those of the PI substrate and Cu_2_O nanoparticles. In addition to Cu diffraction peaks, weak Cu_2_O signals can also be detected. However, those with copper formate additives show much stronger Cu_2_O peaks, which explains the higher electrical resistivity of copper formate-added pastes after sintering.

### 3.2. Changes in the Resistivity of Sintered Copper Thin Films Caused by Bending Deformation

**Figure 6:**
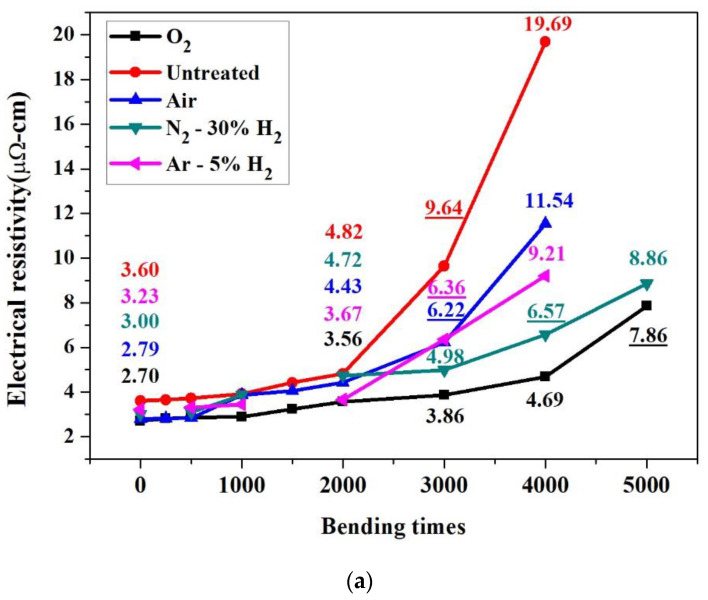

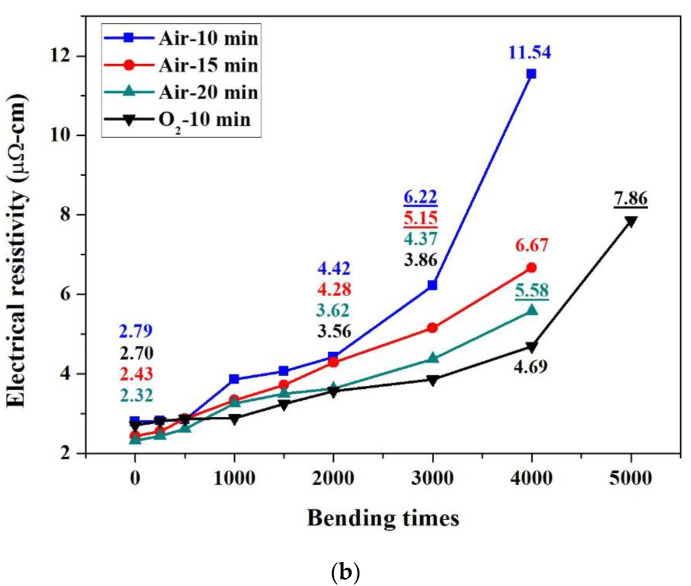
The relationship between the number of bends for test pieces and their respective electrical resistivity: (**a**) substrates modified using different plasmas, and (**b**) substrates modified by O_2_ plasma, and air plasma with different processing periods.

Thin sintered copper conducting layers, with the thickness of about 2~3 μm, formed on PI substrates (each undergoing 10 min bombardment for different plasmas) were subjected to bending tests of up to 5000 bending deformations. The experiment was paused at fixed intervals for the measurement and recording of resistivity. As shown in Figure 6a and Table 1, the initial resistivity of a test piece was 2.7–3.6 μΩ-cm. The resistivity increased slightly as 2000 bends was approached. At 3000 bends, the resistivity of the untreated specimens, those treated in air and in Ar-5%H_2_ conditions increased by more than 100%. A test piece treated with N_2_-30%H_2_ failed after 4000 bends; however, those treated with O_2_ plasma withstood more than 4000 bends. Estimations using interpolation showed that the maximum number of bends withstood for each condition are: 2500 for the untreated specimens, 2700 for the air plasma-treated, 3100 for the Ar-5%H_2_ plasma specimens, 3700 for the N_2_-30%H_2_ plasma condition, and 4200 for the O_2_ plasma specimens.

Considering the effect of plasma processing time, air plasma bombardment was conducted for 10, 15, or 20 min. The data in Figure 6b and Table 1 show that prolonged plasma bombardment certainly improves bending fatigue resistance. However, the bending resistance of test pieces bombarded with air plasma for 20 min was poorer than that provided by O_2_ plasma treatment for 10 min. Again, using interpolation, the maximum number of bends that can be withstood by 10, 15, and 20 min air plasma test pieces were 2700, 2850, and 3300, respectively, which indicated a rather limited improvement.

**Figure 7:**
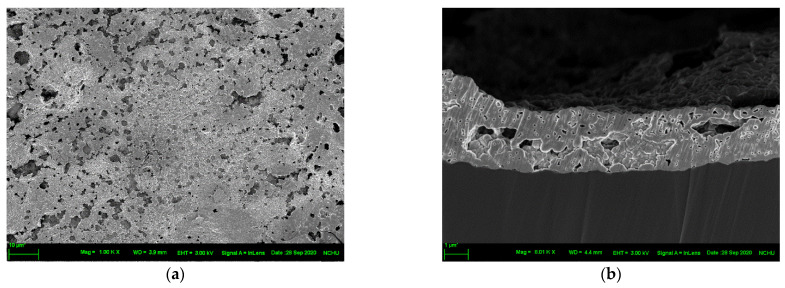

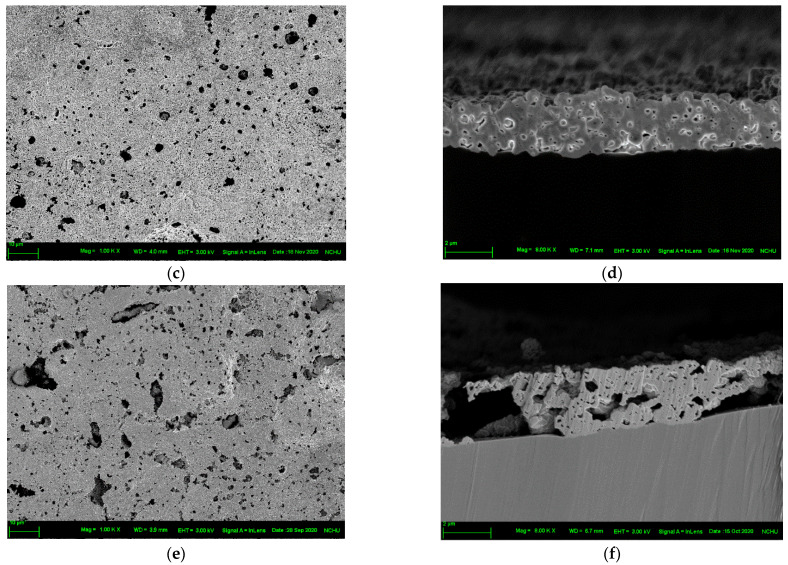
Top view and cross-sectioned images of the sintered structure on untreated PI substrate subjected to different bending cycles: (**a**,**b**) undeformed, (**c**,**d**) 50% of cycles to failure, (**e**,**f**) cycles to failure.

**Figure 8:**
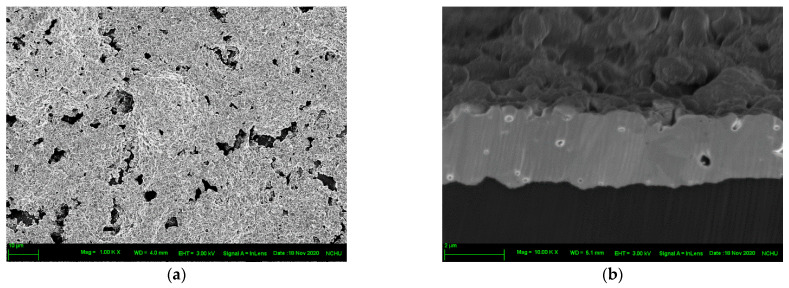

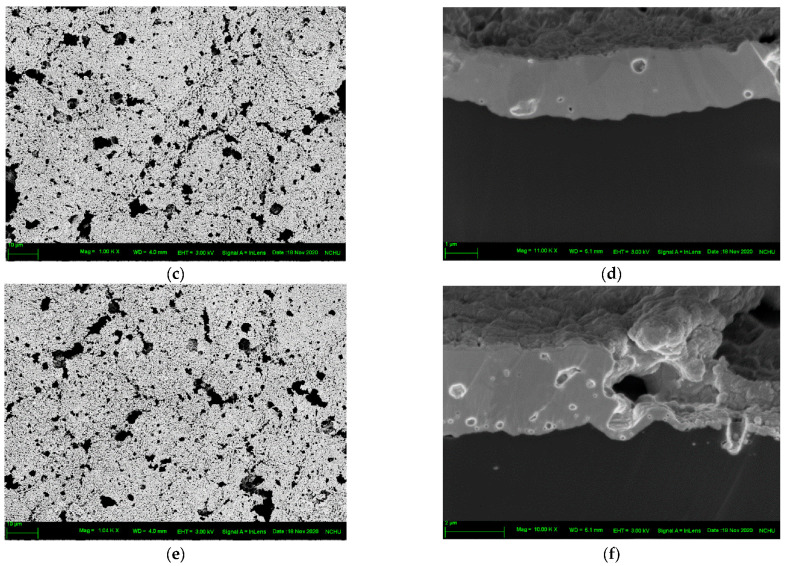
Top view and cross-sectioned images of the sintered structure on O_2_ plasma-treated PI substrate subjected to different bending cycles: (**a**,**b**) undeformed, (**c**,**d**) 50% of cycles to failure, (**e**,**f**) cycles to failure.

Figure 7 and Figure 8 illustrate the top views and cross-sections of the sintered structures on untreated and O_2_ plasma-bombarded PI substrates, respectively, subjected to different bending cycles. In the undeformed state, the surface porosity of sintered copper was 12.9% for untreated specimen, while that for O_2_ plasma specimens was 13.9%. However, it is interesting that the cross-sectioned structures show a significant difference. Untreated specimens reveal a similar porosity with the top view, but the cross-section of O_2_ plasma specimens possessed a much solider structure than the top view. This needs further investigation. For the top view of both the untreated and O_2_ plasma specimens, the pores extended and linked. The porosity for untreated specimens increased from 12.9% (undeformed state) to 14.5% (50% lifetime) to 15.4% (100% lifetime), while that for O_2_ plasma specimens was from 13.9% (undeformed state) to 15.6% (50% lifetime) to 16.3% (100% lifetime). Worthy of notice is that from the images of the cross-sections, the sintered structures detached from the PI substrates and thereby the electrical resistivity rose drastically when the cycles to failure were met.

### 3.3. Surface Properties of PI Substrate after Plasma Modification

**Figure 9:**
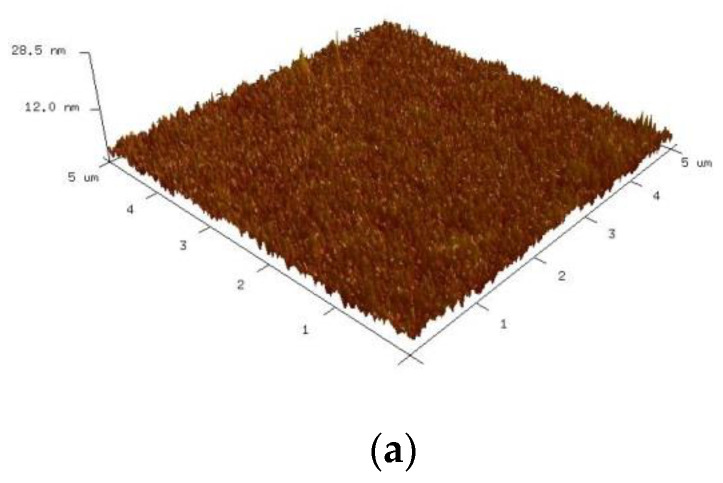

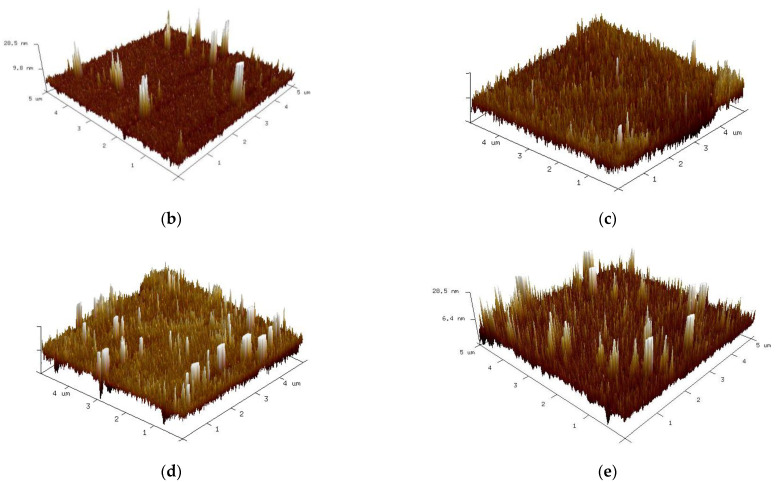
Three-dimensional surface contour diagrams of PI substrates: (**a**) untreated, and treated with different plasmas: (**b**) air, (**c**) Ar-5%H_2_, (**d**) N_2_-30%H_2_, (**e**) O_2_.

The surface roughness of the substrate was measured using atomic force microscopy. The 3D surface contours of the untreated PI substrate and substrate modified by 10 min of bombardment using air, Ar-5%H_2_, N_2_-30%H_2_, or O_2_ gas plasma are presented in Figure 9a–e. The mean surface roughness (R_a_) for each condition was 1.02, 1.69, 1.96, 3.43, and 3.55 nm. A comparison of the mean surface roughness with the maximum number of bends withstood revealed positive correlation. The results indicate that the surface roughness of the substrate contributed to the cohesiveness of the sintered thin film and the substrate, which might in turn enhance fatigue reliability.

**Figure 10:**
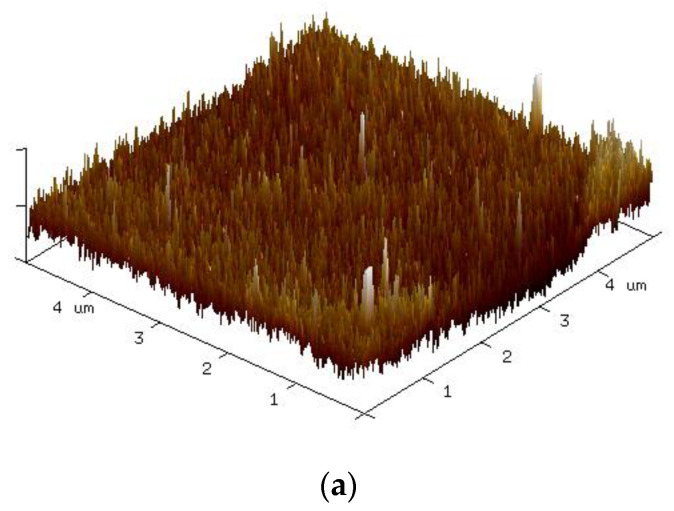

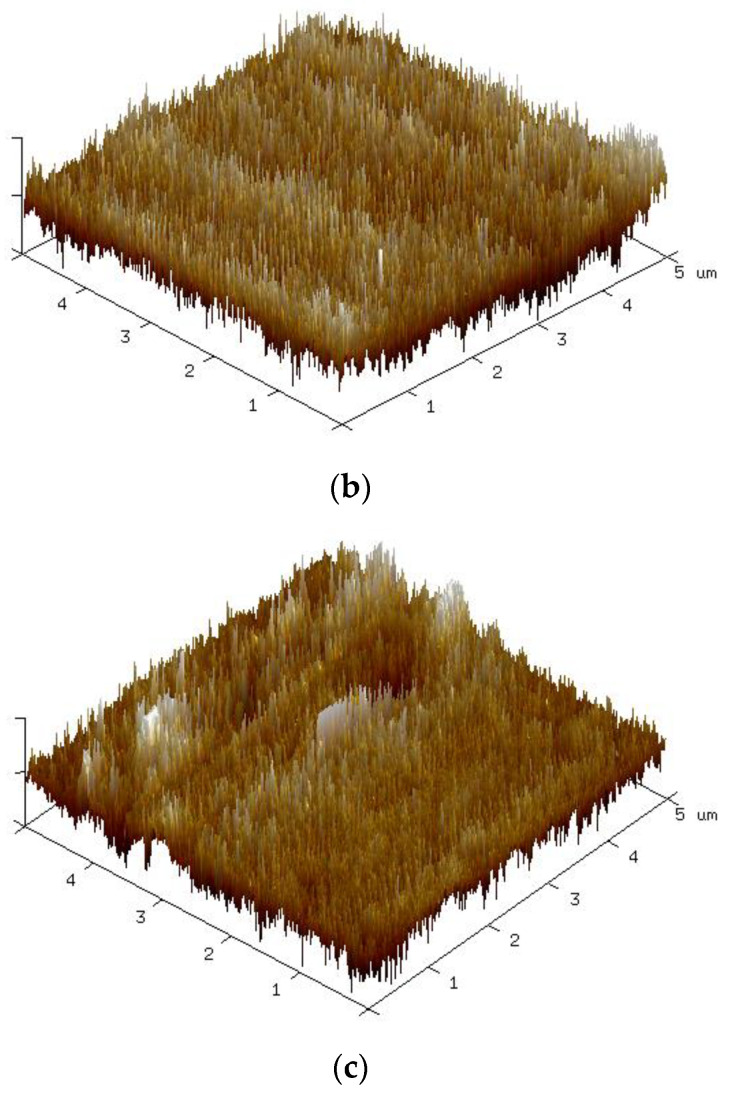
Three-dimensional surface contour diagrams of PI substrates subjected to different durations of air plasma modification: (**a**) 10 min, (**b**) 15 min, and (**c**) 20 min.

Figure 10 shows the 3D surface morphology of the PI substrate modified using bombardment by air plasma for different times. The corresponding surface roughness (R_a_) was 1.69 nm, 2.26 nm, and 2.58 nm at 5, 10, and 20 min, respectively. The surface roughness was also positively correlated with the maximum number of bends withstood. A comparison of Ar-5%H_2_ and air, 15 min test pieces revealed a close maximum number of bends withstood (3100 versus 2850). However, the roughness of these two sets of samples did not show the same tendency (1.96 nm Ar-5%H_2_ and 2.26 nm for air, 15 min). This indicates that surface roughness was not the only factor affecting bending fatigue reliability.

**Figure 11:**
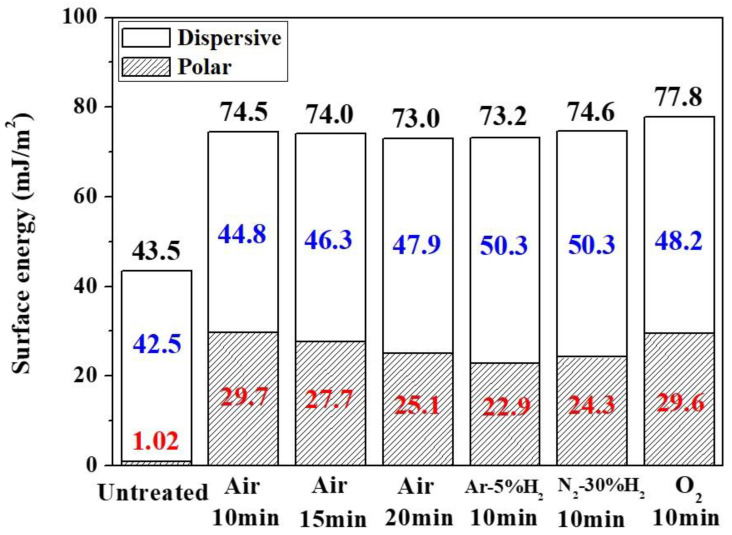
Surface energy of untreated PI substrates and others modified using different gas plasmas.

The contact angle measurement results are shown in Table 2. Substitution of the results into Equations (1) and (2) allowed the surface energy of both the polar and dispersive components to be calculated. The results provided in Figure 11 indicate that regardless of the type of plasma used for the modification, the polar component of the surface energy increased substantially while the increment of the dispersive component remained limited. This indicated that in addition to surface roughness, reaction with plasma caused changes in the surface binding of the PI substrate as well. Figure 11 also revealed that air and O_2_ plasma exhibited a higher polar component increment ratio. From these results it was inferred that oxygen played a key role in the reaction.

### 3.4. Surface Binding Analysis of Plasma-Modified PI Substrate

**Figure 12:**
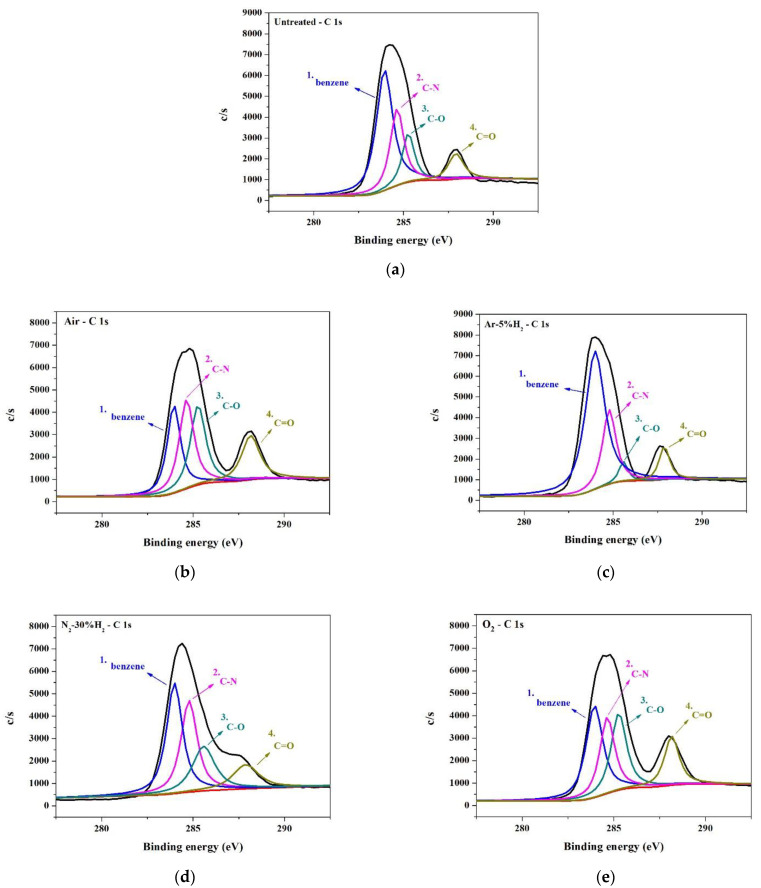
XPS C1s spectra of PI substrate surface: (**a**) untreated, (**b**) air, (**c**) Ar-5%H_2_, (**d**) N_2_-30%H_2_, and (**e**) O_2_.

**Figure 13:**
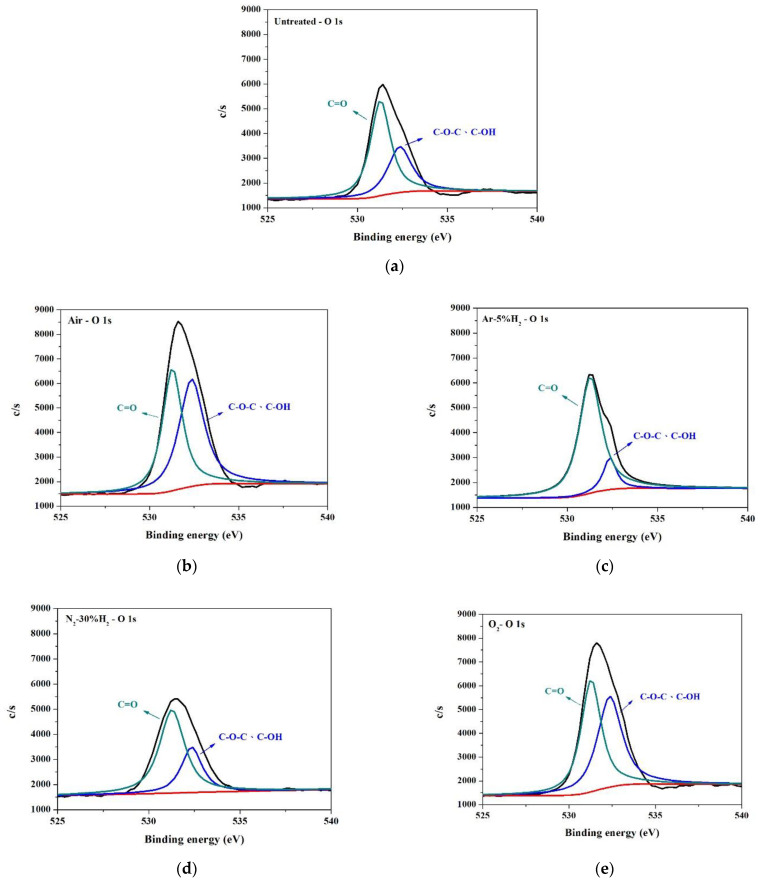
XPS O 1s spectra of PI substrate surface: (**a**) untreated, (**b**) air, (**c**) Ar-5%H_2_, (**d**) N_2_-30%H_2_, and (**e**) O_2_.

**Figure 14:**
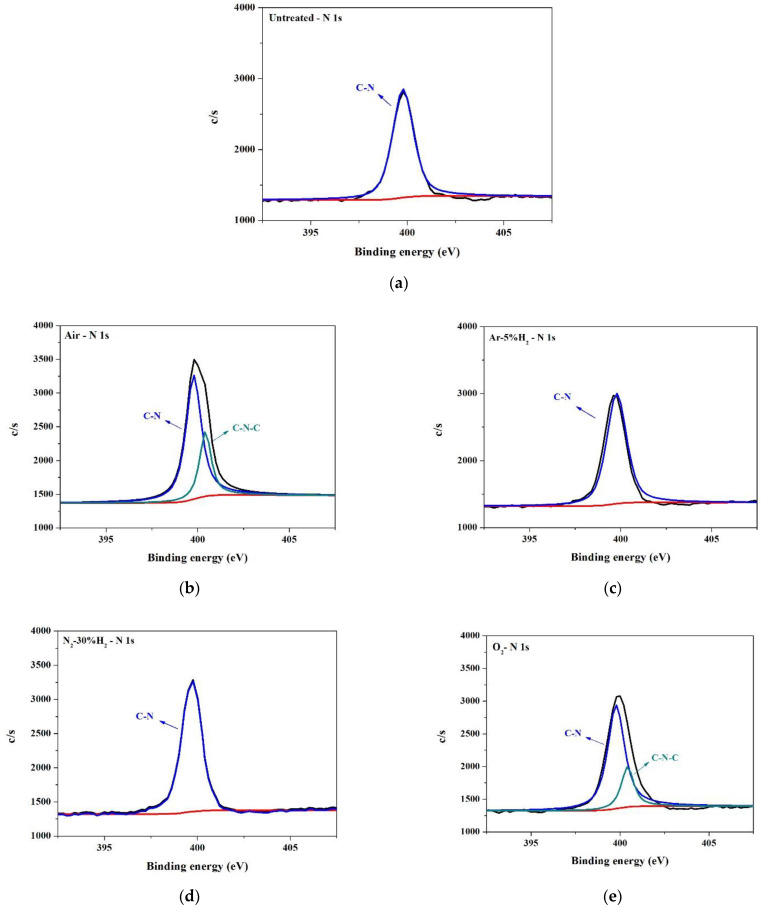
XPS N 1s spectra of PI substrate surface: (**a**) untreated, (**b**) air, (**c**) Ar-5%H_2_, (**d**) N_2_-30%H_2_, and (**e**) O_2_.

Analysis of the surface binding of the PI substrate was carried out using X-ray photoelectron spectroscopy (XPS). The XPS narrow energy spectra of C 1s, O 1s, and N 1s on PI substrates modified using different gaseous plasmas are shown in Figure 12, Figure 13 and Figure 14. As mentioned, the structure of PI can be divided into PMDA and ODA [20]. The C 1s spectrum (Figure 12) can be divided into four peaks, namely, peaks No. 1 to no 4. Table 3 indicates that peak No. 1 was the sum of C-related binding (C=C, C–C, and C–H) on the benzene ring in the ODA structure [25,26]. Peak No. 2 was the sum of C-related binding (C=C, C–C, and C–H) on the benzene ring in the PMDA structure, the C–N bond in the ODA structure, and the C–N–C structure generated after rearrangement [26,27]. Peak No. 3 indicated the presence of three structures, C–O, C–O–C, and C–OH, generated only after plasma modification [26,27]. Lastly, peak No. 4 indicated a single type of binding, namely C=O [26,27]. The O 1s spectrum (Figure 13) has two peaks. One from the combination of C–O–C and C–OH, the other from C=O [25]. The N 1s spectrum (Figure 14) shows C–N and C–N–C.

A comparison of untreated substrate with one modified by O_2_ and air plasma (Figure 13a,b,e and Table 4), shows that after O_2_ containing plasma bombardment (O_2_ and air), the peak values of the C–O–C, C–OH, and C=O groups were notably enhanced. Figure 14a,b,e and Table 5 show that O_2_ containing plasma treatment had no noticeable effect on the intensity of the C-N bonds, but a peak characteristic of C–N–C was present. The above results bring us to believe that modification of the PI substrate surface by O_2_ containing plasma seems to cause the formation of four types of surface binding: C–O–C, C–OH, C=O, and C–N–C.

The H_2_ containing plasma-modified substrates, N_2_-30%H_2_, and Ar-5%H_2_, were also compared with untreated samples. Figure 12a,c,d and Table 3 show that after modification by Ar-5%H_2_ plasma, peak No. 1 exhibited a prominent increment, but this did not happen after treatment with N_2_-30%H_2_ plasma. This suggested that Ar plasma rather than H_2_ caused the increase in C-related surface bindings. Figure 13a,c,d and Table 4 show that treatment with plasma containing H_2_, caused a significant decrease in C–O–C and C–OH, but an increase in C=O. This was especially noticeable with Ar-5%H_2_. A possible reason for this is that C–OH and plasma H^+^ reacted to form C=O and H_2_. Figure 14a,c,d and Table 5 also show that the addition of H_2_ caused a slight enhancement of C–N binding.

Summarizing aforementioned findings, it can be suggested that the superior bending life for the O_2_ plasma-modified samples can be ascribed to two factors: the greatest surface roughness, and the surface bindings thus formed, especially C=O and C–OH, which can enhance the cohesion of PI and metal effectively [10]. To improve the reliability of the sintered copper films on PI substrates, none of them are dispensable.

## 4. Conclusions

In this study, highly conductive copper circuitry was successfully produced by the laser sintering of Cu_2_O composite pastes. The mean resistivity reached was 4 μΩ-cm and lower. Modification of the surface of the PI substrate using different gaseous plasmas increased the resistance of the sintered Cu films to cyclic mechanical bending. The maximum number of bends withstood for each condition were: untreated, 2500; air plasma-treated, 2700; Ar-5%H_2_ plasma-treated, 3100; N_2_-30%H_2_ plasma-treated, 3700; and O_2_ plasma-treated, 4200. The optimal performance was achieved using O_2_ gaseous plasma, which enhanced the bending fatigue threshold by at least 1.68 times. Based on surface properties measurements, one of the main reasons was attributed to that O_2_ plasma treatment causes a roughening of the PI substrate and improves adhesion of the copper. The formation of C=O and C–OH polar bonds on the surface increases the polar component of the surface energy and contributed to the superior performance of the interfacial robustness between the Cu film and the PI substrate. The combined effect causes increased cohesive strength and ultimately results in an improvement of resistance to bending fatigue.

## Figures and Tables

**Table 1:** Electrical resistivity of the sintered structure on various PI substrates subjected to different bending cycles.

	Cycles	0	1000	2000	3000	4000	5000
Conditions							
Untreated		3.6 ± 0.4	3.9 ± 0.5	4.8 ± 0.5	9.6 ± 0.8	19.7 ± 0.6	N/A
Air		2.8 ± 0.3	3.7 ± 0.4	4.4 ± 0.4	6.2 ± 0.5	11.5 ± 0.8	N/A
O_2_		2.7 ± 0.2	2.9 ± 0.3	3.6 ± 0.4	3.9 ± 0.1	4.7 ± 0.4	7.9 ± 0.7
N_2_-30%H_2_		3.0 ± 0.3	3.9 ± 0.3	4.7 ± 0.5	5.0 ± 0.4	6.6 ± 0.6	8.9 ± 0.3
Ar-5%H_2_		3.2 ± 0.3	3.4 ± 0.4	3.7 ± 0.3	6.4 ± 0.3	9.2 ± 0.3	N/A
Air-15 min		2.4 ± 0.2	3.3 ± 0.3	4.3 ± 0.3	5.2 ± 0.3	6.7 ± 0.5	N/A
Air-20 min		2.3 ± 0.2	3.2 ± 0.3	3.6 ± 0.3	4.4 ± 0.3	5.6 ± 0.5	N/A

**Table 2:** Contact angle data.

	Untreated	Air-10 min	Air-15 min	Air-20 min	Ar-5%H_2_	N_2_-30%H_2_	O_2_
Water (polar)	83.7° ± 2.7°	15.1° ± 1.2°	19.0° ± 1.5°	23.7° ± 1.5°	26.2° ± 2.1°	22.5° ± 2.5°	3.6° ± 0.3°
CH_2_I_2_ (non-polar)	34.0° ± 2.1°	28.5° ± 1.7°	24.6° ± 1.6°	19.5° ± 1.3°	8.16° ± 1.1°	8.1° ± 0.9°	18.5° ± 0.9°

**Table 3:** Intensity ratios of signals in the XPS C 1s narrow spectra.

	Untreated	O_2_	Air	N_2_-30%H_2_	Ar-5%H_2_
Peak No. 1 (C=C, C-C, C-H)	49.8%	34.0%	26.2%	36.2%	63.7%
Peak No. 2 (C=C, C-C, C-H C-N, C-N-C)	26.0%	24.8%	29.1%	30.5%	23.8%
Peak No. 3 (C-O, C-O-C, C-OH)	15.8%	24.7%	27.0%	20.5%	4.14%
Peak No. 4 (C=O)	8.3%	16.5%	17.7%	12.8%	8.25%

**Table 4:** Intensity ratios of signals in the XPS O 1s narrow spectra.

	Untreated	O_2_	Air	N_2_-30%H_2_	Ar-5%H_2_
C-O-C C-OH	4231.4 (35.9%)	9421.3 (49.4%)	10730.3 (52.4%)	3590.6 (31.2%)	1613.6 (13.9%)
C=O	7526.9 (64.1%)	9636.5 (50.6%)	9761.3 (47.6%)	7930.2 (68.8%)	9974.4 (86.1%)
Total	11758.3	19057.8	20491.6	11520.8	11588.0

**Table 5:** Intensity ratios of signals in the XPS N 1s narrow spectra (ND: non-detected).

	Untreated	O_2_	Air	N_2_-30%H_2_	Ar-5%H_2_
C-N	2625.4 (100%)	2658.9 (78.2%)	2706.2 (71.9%)	2937 (100%)	2820 (100%)
C-N-C	ND	741.3 (21.8%)	1059.7 (28.1%)	ND	ND

## Data Availability

The raw/processed data required to reproduce these findings cannot be shared at this time due to technical or time limitations.
